# Germline susceptibility variants impact clinical outcome and therapeutic strategies for stage III colorectal cancer

**DOI:** 10.1038/s41598-019-40571-0

**Published:** 2019-03-08

**Authors:** Peng-Chan Lin, Yu-Min Yeh, Pei-Ying Wu, Keng-Fu Hsu, Jang-Yang Chang, Meng-Ru Shen

**Affiliations:** 1 Department of Internal Medicine, National Cheng Kung University, Tainan, Taiwan; 2 Graduate Institute of Clinical Medicine, National Cheng Kung University, Tainan, Taiwan; 3 Department of Obstetrics and Gynecology, National Cheng Kung University, Tainan, Taiwan; 4 Department of Computer Science and Information Engineering, College of Electrical Engineering and Computer Science, National Cheng Kung University, Tainan, Taiwan; 5 National Institute of Cancer Research, National Health Research Institutes, Tainan, Taiwan; 6 Department of Pharmacology, National Cheng Kung University Hospital, College of Medicine, National Cheng Kung University, Tainan, Taiwan

## Abstract

Although somatic mutations are the main cause of cancer, underlying germline alterations may affect cancer outcome. There is little information on comprehensive analysis of germline genome sequencing for cancer healthcare strategy. Here we studied the implication of germline cancer-associated variants on cancer counselling and therapeutic strategies by germline whole genome and tumor targeted sequencing. Fifty-five gynecological and 104 colorectal cancer (CRC) patients were enrolled. We identified 22 germline pathogenic variants in 16 cancer-associated genes. Most of them are involved in DNA repair signaling, including *MLH1*, *BRCA1/2*, *MUTYH*, *ATM*, *PMS2*, *MSH6*, *BAP1*, and *FANCA*. About 6% of cancer patients presented the secondary findings of germline variants with non-oncogenic impact, mainly on the cardiovascular system which should be carefully monitored during chemotherapy. CRC patients carrying germline susceptibility variants had better disease-free survival than those without variants. Importantly, in the CRC model, the underlying germline alterations mold the tumor somatic alteration landscape. *NOTCH1* mutation was the most common somatic mutation in recurrent CRC, implying a potential therapeutic target in adjuvant setting. In conclusion, both tumor genome and germline sequence data have to be analyzed to have a more complete picture of the overall genetic foundation of cancer.

## Introduction

Genomic medicine has been widely applied in the diagnosis, treatment, and prevention of cancer. The identification of critical genetic mutations is an important diagnostic criterion for certain types of cancer, such as the *BCR-ABL* fusion gene in chronic myeloid leukemia^1^. In cancer treatment, detecting recurrent oncogenic mutations has become standard practice. Currently, *EGFR*, *ALK*, and *ROS1* mutations in non-small cell lung cancer^2^, *RAS* and *BRAF* mutations in colorectal cancer^3^, and *BRAF* mutations in melanoma are routinely used in clinical practice^4^. The individual differences resulting in distinct treatment outcome and toxicity are becoming importance. The Clinical Pharmacogenetics Implementation Consortium (CPIC) therefore recommends analyzing patient genetic polymorphisms before chemotherapy to avoid excess toxicities, such as *UGT1A1* genotyping when prescribing irinotecan^5^.

Advances in next generation sequencing (NGS) have led to the identification of hundreds of mutated genes, including single nucleotide variations (SNVs), copy number variations (CNVs), small insertion/deletions (indels), and fusion genes in a single assay. Mutations in germline cancer susceptibility genes may contribute to hereditary cancer risks. Targeted multigene panels for testing inherited cancer susceptibility are also applied in the diagnosis of hereditary cancer predisposition^6,7^. As technical barriers and genome sequencing costs decrease, whole genome sequencing (WGS) and whole exome sequencing (WES) are increasingly used to establish the mutation landscape of diseases^8,9^. However, there is a lack of comprehensive analysis of germline genetic variants associated with increased cancer susceptibility combined with different treatment responses and drug-related toxicity. Moreover, germline WGS can detect pathogenic variants in the secondary finding genes recommended by the American College of Medical Genetics and Genomes (ACMG), which likely have an impact during cancer treatment.

For patients with a family history of cancer or genetic predisposition to cancer, appropriate risk assessment, accurate detection of causal genes, and proper cancer treatment and surveillance are all crucial, not only for patients themselves but also for their families. Cancer susceptibility genes may affect different molecular signal pathways in cancer formation which impact distinct responses to treatments^10^. Various targeted cancer panels have been developed and clinically applied to identify somatic mutations in genes or pathways that can be targeted therapeutically^11,12^. However, clinically favorable responses to actionable mutations with matched therapy are limited because there are few FDA-approved companion therapies, ambiguous scientific contexts, and loose treatment algorithms^13^. Reviewing these clinical trials^14,15^ with a molecular profiling approach indicates that the treatment algorithms for selecting matched molecularly-targeted agents might be inadequate, and knowing the underlying mechanism of biological pathways may be beneficial to the outcome of advanced or refractory cancer patients.

Here we utilized the germline WGS and tumor deep targeted sequencing to identify germline susceptibility variants that may impact the clinical outcome and therapeutic strategies. We also highlighted the secondary genomic findings on non-oncogenic phenotypes associated with life-threatening adverse events during cancer treatment.

## Materials and Methods

### Patient and normal healthy subject enrollment and counseling

All methods of this study were performed in strict accordance with the relevant guidelines and regulations. The National Cheng Kung University Hospital institutional review board approved this study (A-ER-103-395 and A-ER-104-153), and all participants gave informed written consent. A total of 104 colorectal (CRC), 31 ovarian, and 24 endometrial cancer patients were recruited as the study group in National Cheng Kung University Hospital (NCKUH) between January 2015 and March 2017. Follow-up continued through March, 2018. The follow-up period is every three months until progression or death. (Supplementary Table 1). All CRC patients were pathological stage III and received standard surgical resection followed by adjuvant chemotherapy with the regimen of mFOLFOX6 (5-fluorouracil, leucovorin, and oxaliplatin). For endometrial or ovarian cancer, carboplatin plus paclitaxel were used as post-operative adjuvant chemotherapy. Clinical information, including detailed cancer family history and blood sampling for WGS, was collected at the time of enrollment. WGS, health, and lifestyle data of 499 non-cancer normal Taiwanese people were obtained from Taiwan Biobank as a reference group.

### Germline whole genome sequencing

Whole blood was collected for genomic DNA extraction. Genomic DNA was quantified with a Qubit fluorescence assay (Thermo Fisher Scientific) and sheared with a S2 instrument (Covaris). Library preparation was carried out using the TruSeq DNA PCR-Free HT kit (Illumina). Individual DNA libraries were measured by 2100 Bioanalyzer (Agilent) qPCR and Qubit (Thermo Fisher Scientific). Normalized DNA libraries were combined into five-sample pools per flow cell in all eight lines and clustered on a cBot instrument (Illumina) with Paired-End Cluster Kit V4 (Illumina). All flow cells were sequenced on the HiSeq. 2500 sequencer (Illumina) using SBS kit V4 chemistry (Illumina). FastQC was used to check read quality, and the resulting reads were aligned to the hg19 reference genome with the BWA-MEM algorithm^16^. SNV and indel identification and genotyping were performed across all samples simultaneously using standard hard filtering parameters or variant quality score recalibration according to GATK Best Practices recommendations^17,18^. WGS was performed with a minimum median coverage of 30X.

### Targeted tumor sequencing by cancer panel

A total of 107 primary tumor samples were sent for histologic assessment followed by extraction of nucleic acids from formalin-fixed paraffin-embedded blocks in NCKUH. Specimens were reviewed by pathologists who determined the percentage of viable tumor nuclei and adequacy for profiling mutation detection. Tumor deep targeted sequencing was performed by Oncomine Comprehensive Assays (OCA) (Thermo Fisher Scientific)^19^. The OCA was designed to detect 143 solid tumor-related genes, including 73 hotspot genes, 49 focal CNV gains, 26 genes for full coding region sequencing (CDS), and 22 fusion driver genes. The Ion PGM Sequencing 200 Kit v.2 was used with the Ion PGM sequencer (Thermo Fisher Scientific) according to the manufacturer’s instructions. All samples were analyzed using the Torrent Suite Software 5.0.4, aligning all reads to the hg19 reference genome, and variant calling was performed running the Torrent Variant Caller plugin version 5.0.4.0.

### Genetic susceptibility variants selected for analysis

We used the ANNOVAR^20^ tool to annotate SNVs and indels and filter out variants reported in the 1000 Genomes Project, the Single Nucleotide Polymorphism Database (dbSNP), and allele frequency cutoffs above 0.1% in the Exome Aggregation Consortium (ExAc) database^21^. We chose definitions to select for germline genetic susceptibility variants, including germline cancer-associated genes, drug response genes, and ACMG secondary finding genes. Cancer-associated genetic variants classified as pathogenic variants according to the review of experts were identified and then filtered by our in-house control, the database from Taiwan Biobank. Clinical significance and classification criteria of genetic variants are defined by National Center for Biotechnology Information (NCBI)-ClinVar, a freely available curated database of relationships among medically important genetic variants and phenotypes^22,23^. The 565 well-known cancer-associated genes reviewed by a previous study^24^ and genetic variants associated with the cancer phenotype sumarized in the ClinVar database were selected for analysis. The Pharmacogenomics Knowledgebase (PharmGKB) provides information about the impact of human genetic variation on drug responses^25^ and is used for selecting drug response and adverse drug reaction genetic variants found among chemotherapy-related genetic variants. The updated ACMG secondary findings recommendations were used to identify genetic variants with important non-oncogenic phenotypes^26^. The analytic workflow of drug response and ACMG genetic variants includes automatic extraction matching and database annotation (Supplementary Fig. 1).

### Statistical analysis

Chi-Square test, Fisher’s exact test, and unpaired *t*-test were used to assess the difference between groups. Kaplan–Meier curves were used to assess disease-free survival. Disease-free survival was defined as the time between surgery and recurrence of cancer. A *P*-value < 0.05 was considered statistically significant.

## Results

### Study flow chart and genomic findings on potential life-threatening adverse events

**Figure 1:**
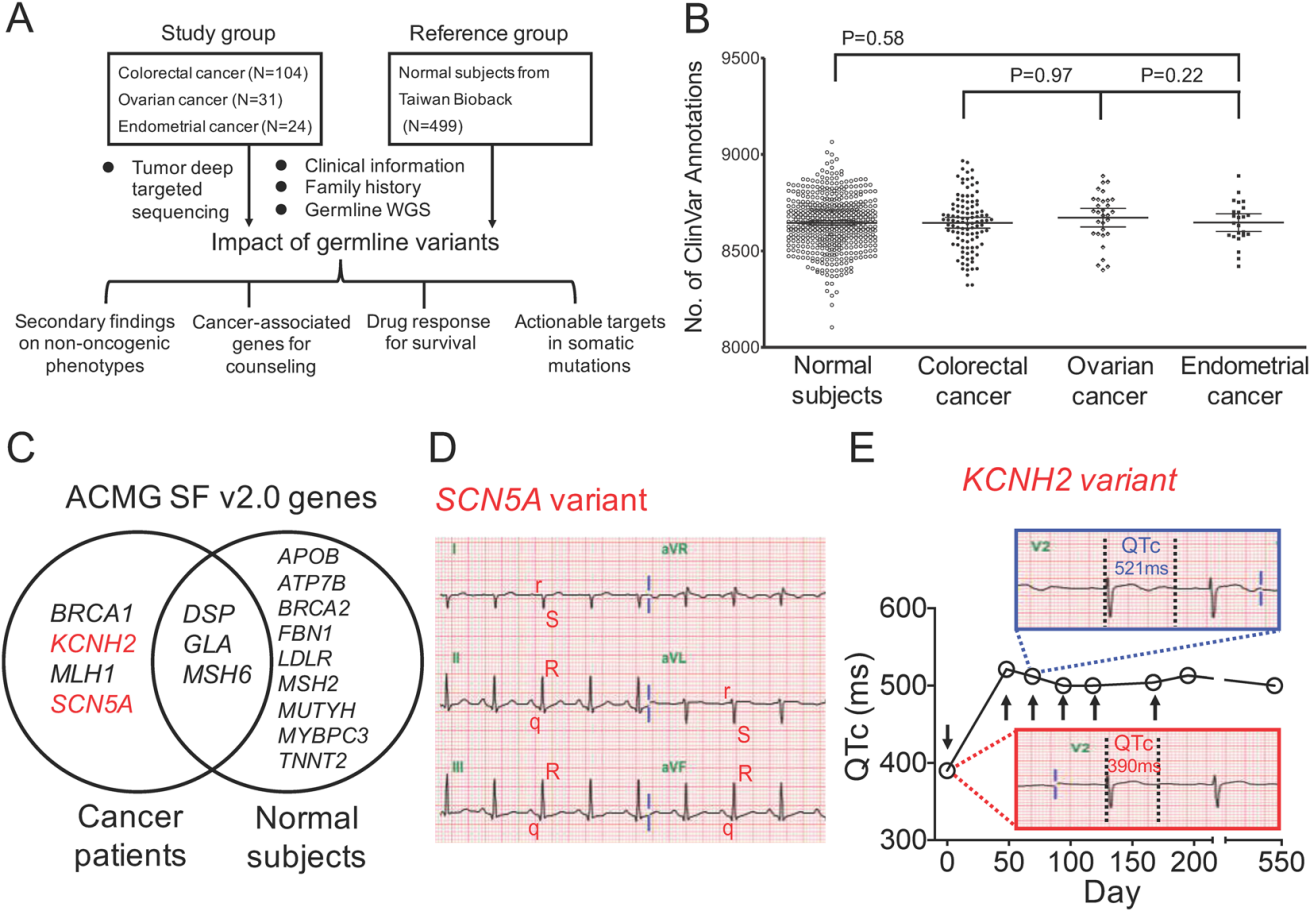
Study flow chart and the genomic findings on potential life-threatening adverse events during chemotherapy. (**A**) Flowchart of WGS and tumor deep targeted sequencing analyses for the identification of germline genetic variants and their implications on patient outcomes. A total of 159 cancer patients and 499 normal subjects were enrolled. WGS data generated from germline DNA were used to analyze the presence of single-nucleotide variants (SNVs) and small insertions and deletions (indels). Identified genetic variants were correlated to clinical information to determine the impacts of germline variants. **(B)** Numbers of ClinVar annotation in the normal population, colorectal, ovarian, and endometrial cancer patients. Each genetic variant was compared with published alterations in the ClinVar database. The number of genetic variants with ClinVar annotation was compared between the normal population and different types of cancers by *t*-test. **(C)** ACMG SF v2.0 genes with pathogenic variants in 159 cancer patients and 499 normal subjects. Germline genetic variants were compared with the list of genes recommended by ACMG to be reported as incidental or secondary findings (SFs). ACMG SF v2.0 genes with pathogenic variants detected in cancer patients and normal subjects were listed in the left and right circle, respectively. **(D)** The EKG shows right axis deviation, small R waves with deep S waves (rS complexes) in leads I, and aVL and small Q waves with tall R waves (qR complexes) in II, III, and aVF, suggesting left posterior fascicular block. **(E)** Corrected QT (QTc) interval in an endometrial cancer patient at baseline and after the first cycle of treatment with carboplatin and paclitaxel. Changes in QTc interval before, during, and after the six cycles of chemotherapy. EKG was performed at baseline (before surgery), before each cycle of carboplatin plus paclitaxel, and one year after chemotherapy. Arrows indicate the timing of surgery and chemotherapy, respectively.

Figure 1A shows the study design, in which 159 cancer patients and 499 normal healthy subjects were enrolled as the study and reference groups, respectively. In the study group, 104 (65%) patients were CRC and 55 (35%) patients were gynecological cancer (Supplementary Table 1). Forty percentage of cancer patients presented the family history of cancer in the first-degree relatives, and 12% of them showed cancer family history in the second-degree relatives. In terms of ClinVar annotation, 1,369,942 genetic variants (SNVs and small indels) were annotated in 159 cancer patients. A total of 48,419 genetic variants (reviewed by experts), including pathogenic, uncertain significance, drug response, benign, and likely benign categories, were identified.“Reviewed by experts” indicates that the evidence to support the interpretation of genetic variants is provided by a ClinGen-approved expert panel^23^. There were 8,323 to 8,967 genetic variants (median: 8,661) per cancer patient and 8,104 to 9,065 genetic variants (median: 8,649) per normal subject (Fig. 1B). There was no significant difference in ClinVar annotation numbers between cancer patients and normal subjects.

Germline genetic variants associated with non-oncogenic phenotypes were first analyzed by comparing with ACMG secondary findings (SF v2.0) gene list^26^. We identified 12 ACMG SF v2.0 genetic variants in 23 of 499 normal subjects (4.6%), and 7 genetic variants were identified in 9 of 159 cancer patients (5.6%) (Fig. 1C, Supplementary Table 2), including *BRCA1*, *KCNH2*, *MLH1*, *SCN5A*, *DSP*, *GLA*, and *MSH6*. Four of these 7 genetic variants, including *KCNH2*, *SCN5A*, *DSP*, and *GLA*, were associated with cardiovascular diseases. For instance, *SCN5A* is associated with Brugada syndrome-1 (MIM 601144)^27^. The *SCN5A* variant was identified in a 56-year-old patient with ovarian cancer. This patient did not have past history of heart disease. Although no typical manifestation of Brugada syndrome was observed, the EKG demonstrated right axis deviation, rS complexes in leads I and aVL, and qR complexes in leads II, III, and aVF, which suggested left posterior fascicular block (Fig. 1D). *KCNH2* is associated with long QT syndrome 2 (MIM 613688)^27^. One 63-year-old patient with endometrial cancer had the c.1714G > A (p.Gly572Ser) variant in *KCNH2*. This patient received the standard surgical resection followed by adjuvant chemotherapy with the regimen of carboplatin and paclitaxel for 6 cycles. The baseline EKG, which was performed before surgery, showed normal sinus rhythm with the QTc around 390 ms (Fig. 1E). After the first cycle of chemotherapy, the QTc interval significantly increased from 390 to 521 ms (Fig. 1E). This patient received a total of 6 cycles of chemotherapy. The QTc interval was around 500–515 ms during the period of chemotherapy and the prolonged QTc was still noted 1 year after the completion of chemotherapy (Fig. 1E). We have reviewed the family history for the individuals with “incidental findings”. There are no associated diseases with “incidental findings” in the first-degree and the second-degree relatives. These findings highlight the importance of identifying germline variants associated with non-oncogenic phenotypes which should be carefully monitored during cancer treatment.

### Ethnic difference of germline susceptibility variants

Identifying germline mutations in cancer susceptibility genes was important for genetic counseling. The ethnic difference in the genomic background should be taken into consideration to minimize false-positive results. Based on the public dataset^20^, 46 candidates of germline genetic variants associated with cancer phenotypes were identified in the study and reference group (Supplementary Table 3). The top 10 genetic variants with the frequency ≥1% detected in the reference group were listed in Table 1. In the database of Genotypes and Phenotypes (dbGaP)^28^, the frequency of *PRSS1* c.161A > G, *SLC22A18* c.257G > A, and *RAD54B* c.1778A > G was 0.42, 1.35, and 0.86%, respectively. Contradictorily, these three genetic variants had relatively high frequency in this study (Table 1), indicating that the pathogenicity of these variants for cancer was less likely in Taiwanese population. Therefore, these three genetic variants could be misclassified as pathogenic variants in ClinVar database.

**Table 1:** In-house filtering for cancer-associated genetic variants.

Gene	Name	Cancer patients (%)	Normal subjects (%)	dbGaP (%)
FGFR4	FGFR4:c.1162G > A (p.Gly388Arg)	71.07	69.14	45.8
PTPRJ	PTPRJ:c.827A > C (p.Gln276Pro)	49.06	48.70	26.9
PRSS1	PRSS1:c.161A > G (p.Asn54Ser)	29.56	32.46	0.42
SLC22A18	SLC22A18:c.257G > A (p.Arg86His)	16.35	12.42	1.35
PRSS1	PRSS1:c.47C > T (p.Ala16Val)	13.21	10.62	7.18
EHBP1	EHBP1:c.1185 + 30064G > A	11.32	5.81	NA
RAD54B	RAD54B:c.1778A > G (p.Asn593Ser)	1.26	3.01	0.86
MLH1	MLH1:c.649C > T (p.Arg217Cys)	1.26	1.00	NA
TEK	TEK:c.2228G > C (p.Gly743Ala)	0.63	1.00	NA
MSH6	MSH6:c.4001 + 2_4001 + 5delTAAC	0.63	1.00	NA

Based on the public dataset, candidates of germline genetic variants associated with cancer phenotypes were identified in cancer patients and normal subjects. The top 10 genetic variants with the frequency ≥1% detected in normal subjects were listed. N the dbGaP, the frequency of *PRSS1* c.161A > G, *SLC22A18* c.257G > A, and *RAD54B* c.1778A > G was 0.42, 1.35, and 0.86%, respectively. On the other hand, these three genetic variants had relatively high frequency in our cancer patients and normal subjects, indicating that the pathogenicity of these variants for cancer was less likely in Taiwanese population. dbGaP: the database of Genotypes and Phenotypes; NA: not available.

### Identification of cancer-associated genetic variants for genetic counseling

**Figure 2:**
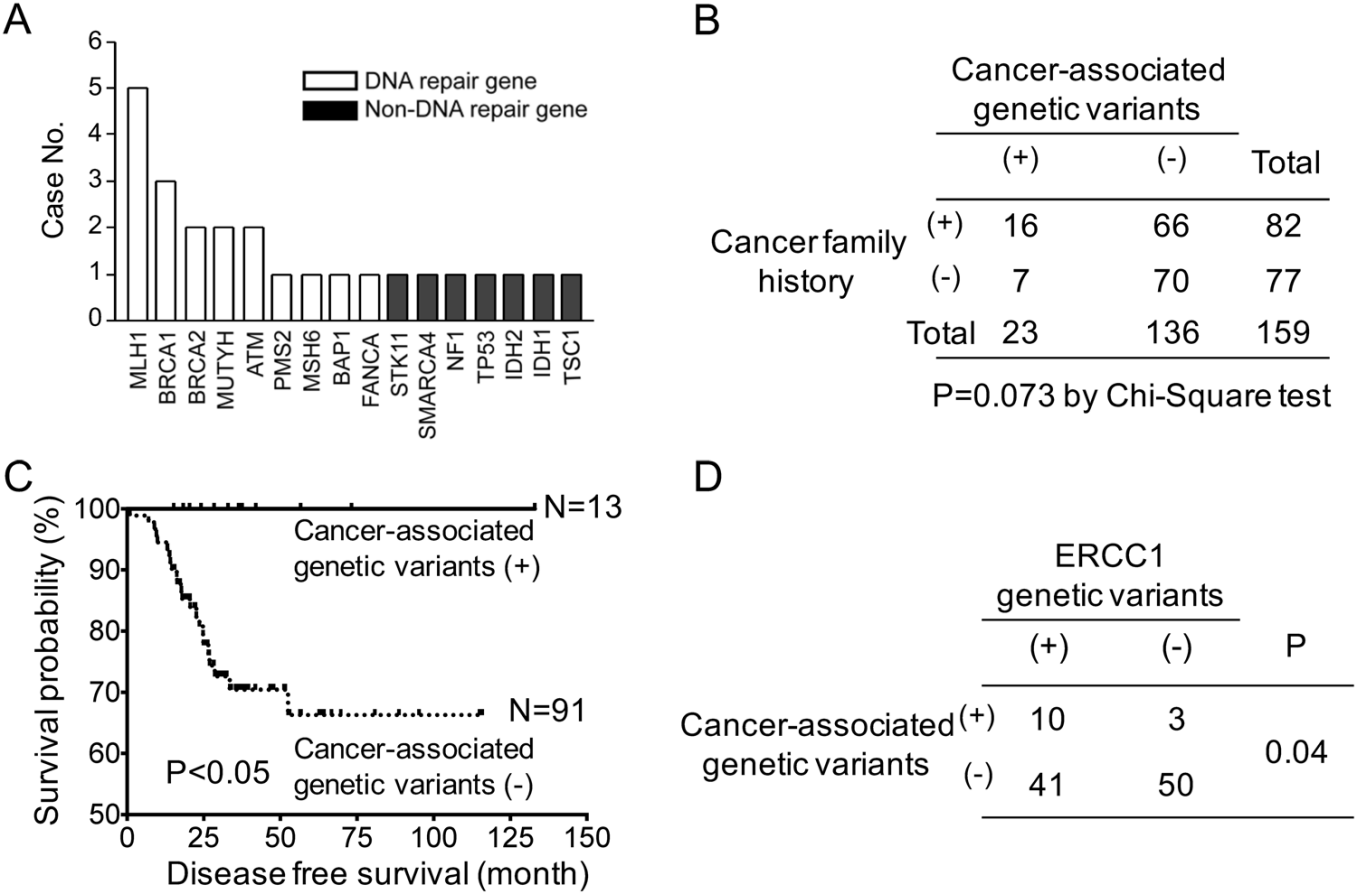
The impact of germline cancer-associated variants on clinical outcome. **(A)** Germline cancer-associated variants identified in 55 gynecological and 104 CRC patients. Variants of cancer-associated genes in the ClinVar database were selected for analysis. After filtering out in-house control, 16 germline cancer-associated variants were detected in 23 patients. **(B)** The relationship between germline cancer-associated variants and cancer family history tested by Chi-square test. **(C)** The Kaplan-Meier curves of disease-free survival in CRC patients with and without germline cancer-associated variants. **(D)** The relationship between germline cancer-associated variants and drug response *ERCC1* gene tested by Chi-square test.

After filtering out in-house control, 22 pathogenic variants in 16 cancer-associated genes were detected in 23 of 159 patients, including 13 (12.5%) CRC patients and 10 (18.2%) gynecologic cancer patients (Supplementary Table 4). Five patients had pathogenic variants in *MLH1*, which was the most common gene harboring a germline mutation in the study group. Other pathogenic variants were in *BRCA1*, *BRCA2*, *MUTYH*, *ATM*, *PMS2*, *MSH6*, *BAP1*, and *FANCA*, which were DNA repair-associated genes (Fig. 2A). We also detected the pathogenic variants in *STK11*, *SMARCA4*, *NF1*, *TP53*, *IDH1*, *IDH2*, and *TSC*. These genes were non-DNA repair-associated genes, and the frequency of pathogenic variants in these genes was relatively low. These results suggested that the defects in DNA repair pathways contribute to carcinogenesis and are potentially attractive targets for therapy, such as PARP inhibitors^29^.

We studied the association between cancer family history and germline mutations of cancer-associated genes. As shown in Fig. 2B, the incidence of germline cancer-associated genetic variants is 19.5% for patients with cancer family history, whereas the incidence is decreased to 9.1% for patients without history. There is a trend showing the correlation between the germline cancer-associated genetic variants and cancer family history in spite of non-statistical significance.

The hereditary cancer syndromes were diagnosed in three patients. Two patients harboring the *MLH1* mutation had the diagnosis of Lynch syndrome (Supplementary Fig. 2A,B). One patient had the germline variant *BRCA1* c.122A > G confirmed by Sanger sequencing. Her mother was diagnosed with ovarian cancer at the age of 50 (Supplementary Fig. 2C). However, among 23 patients with germline cancer-associated genetic variants, 7 patients did not show the family history of cancer (Fig. 2B and Supplementary Fig. 2D). Although we did not detect any germline cancer-associated variants in the other 136 patients, 66 of these patients (49%) still had a family history of cancer (Fig. 2B and Supplementary Fig. 2E).

### Impact of germline susceptibility variants on survival

The association between cancer susceptibility variants and clinical outcome is uncertain. Here, we observed that CRC patients carrying cancer-associated genetic variants had better disease-free survival than those without variants (*P* < 0.05, Fig. 2C). All CRC patients in the study group were pathological stage III and received adjuvant chemotherapy with mFOLFOX6. Previous studies showed that the excision repair cross-complementing (*ERCC1*) variants were a predictive marker of oxaliplatin-based chemotherapy^30^. Accordingly, we studied the incidence of *ERCC1* genetic variants in CRC cancer. As shown in Fig. 2D, 77% of CRC patients with cancer-associated variants had the *ERCC1* c.354T > C variant. In contrast, 45% of CRC patients without cancer-associated variants had *ERCC1* variant (*P* = 0.04). The higher percentage of *ERCC1* variant in CRC patients with pathogenic variants of cancer-associated genes might explain the better survival in this group of patients.

### Actionable targets of somatic mutations

**Figure 3:**
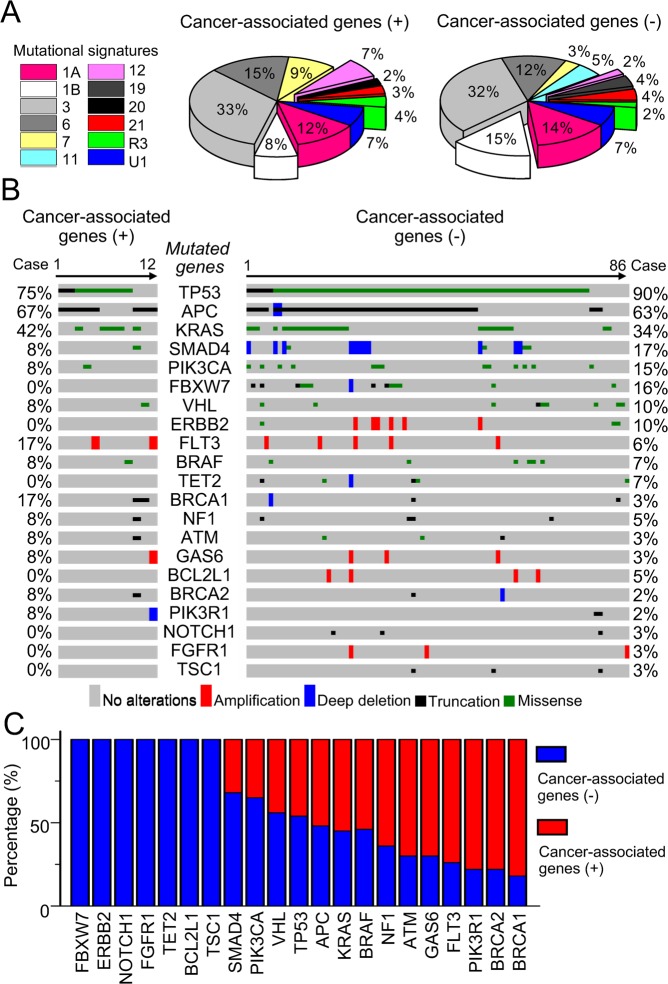
Underlying germline cancer-associated variants mold the somatic alteration landscape. (**A**) Distribution of mutational signatures in CRC patients with or without cancer-associated genetic variants. Different colors indicate different types of mutational signatures. **(B)** The genomic landscapes of tumor somatic mutations in CRC patients with (n = 12) or without (n = 86) germline cancer-associated genetic variants. **(C)** Dominant somatic mutations in different germline backgrounds. Somatic mutations in Notch signal pathways (*FBXW7* and *NOTCH1*) or receptor tyrosine kinase signaling (*ERBB2*, *FGF1R*) exclusively happened in CRC patients without germline cancer-associated variants. Somatic mutations in DNA repair signalingj (*ATM*, *BRCA1*, *BRCA2*) were mainly noted in CRC patients carrying germline cancer-associated variants.

Compared to patients with cancer-associated genetic variants, the disease-free survival of patients without variants was shorter (Fig. 2C). We hypothesized the underlying germline alterations may mold the somatic alteration landscape and cooperate with acquired mutations to promote tumor onset and maintenance. To identify actionable somatic mutations perhaps provides opportunities to improve the clinical outcome of these patients. Therefore, we performed deep targeted sequencing analysis of 98 available CRC primary tumor samples. Twelve types of tumor mutational signatures^31^ were observed in 98 CRC patients (Fig. 3 and Supplementary Fig. 3). The tumor mutational signature 1A, 3, 6, 7, 21, R3 and U1 contributed similarly between patients with or without germline cancer-associated variants. In contrast, the frequency of mutational signature 1B, 11, 12 and 19 was significantly different between two groups of patients. Signature 1B, which is an age-associated mutation signature, accounting for 8% and 15% of patients with and without germline cancer-associated variants, respectively. Moreover, tumor mutational signature 11 and 19 were exclusively observed in patients without carrying germline cancer-associated variants and mutational signature 20 could only be found in patients carrying germline cancer-associated variants. These results suggest that the underlying germline alterations indeed mold the types of tumor mutational signature.

Moreover, 434 somatic mutations in 48 genes were detected in 98 primary tumor samples (Fig. 3B). *TP53*, *APC* and *KRAS* were the most frequently mutated genes detected in patients with and without germline cancer-associated variants. In contrast, *FLT3*, *BRCA1*, *BRCA2* and *PIK3R1* mutations were more frequently detected in patients carrying germline cancer-associated variants (Fig. 3B,C). On the other hand, the frequency of *SMAD4* and *PIK3CA* mutation was higher in patients without germline cancer-associated variants (17% and 15%, respectively) than those with variants (8% and 8%, respectively). Moreover, mutations of *FBWX7*, *ERBB2*, *TET2*, *BCL2L1*, *NOTCH1*, *FGFR1* and *TSC1* were only detected in patients without germline cancer-associated variants (Fig. 3B,C). Mutations of *FBXW7* and *NOTCH1* lead to aberrant activation of Notch signaling pathway. *ERBB2* and *FGFR1* are well-known receptor tyrosine kinases involved in the carcinogenesis of different types of cancers. These results imply that patients with and without germline cancer-associated variants may harbor different tumor somatic mutations. Notch, ERBB2, and FGFR1 signaling pathway might be potential therapeutic targets to improve the outcome of CRC patients without germline cancer-associated genetic variants in adjuvant setting.

### Emerging NOTCH1 mutated subclones in tumor recurrence

To identify potential therapeutic targets, we analyzed the somatic mutations of primary and recurrent tumor samples from 9 CRC patients with recurrent diseases. By comparing the somatic mutations between primary and recurrent tumors, we identified the mutated genes only detected in recurrent tumor, including *NOTCH1*, *RAC1*, *MLH1*, *TSC1*, *PTCH1*, *NF1*, *ERBB3*, *DNMT3A*, *CDH1*, and *PIK3R* (Table 2). The most common mutated gene detected in recurrent tissue was *NOTCH1* gene, which was identified in 7 of 9 CRC patients.

**Table 2:** Protein-coding mutated genes identified in primary–recurrent tumor pairs.

Patient	Total exonic mutations (Primary/recurrent)	Recurrent-specific somatic mutation genes
1	19/20	NOTCH1, RAC1, VHL
2	25/23	RB1, MLH1, NOTCH1, TSC1
3	33/23	PTCH1, NF1
4	19/20	NOTCH1
5	14/18	ERBB3, DNMT3A, NOTCH1
6	23/21	DNMT3A, CDH1
7	22/21	DNMT3A, NOTCH1, PIK3R1
8	22/20	NOTCH1
9	19/17	NOTCH1

The somatic mutations of primary and recurrent tumor samples from 9 CRC patients were analyzed. By comparing the somatic mutations between primary and recurrent tumors, the mutated genes only detected in recurrent tumor, including NOTCH1, RAC1, MLH1, TSC1, PTCH1, NF1, ERBB3, DNMT3A, CDH1, and PIK3R, were identified.

**Figure 4:**
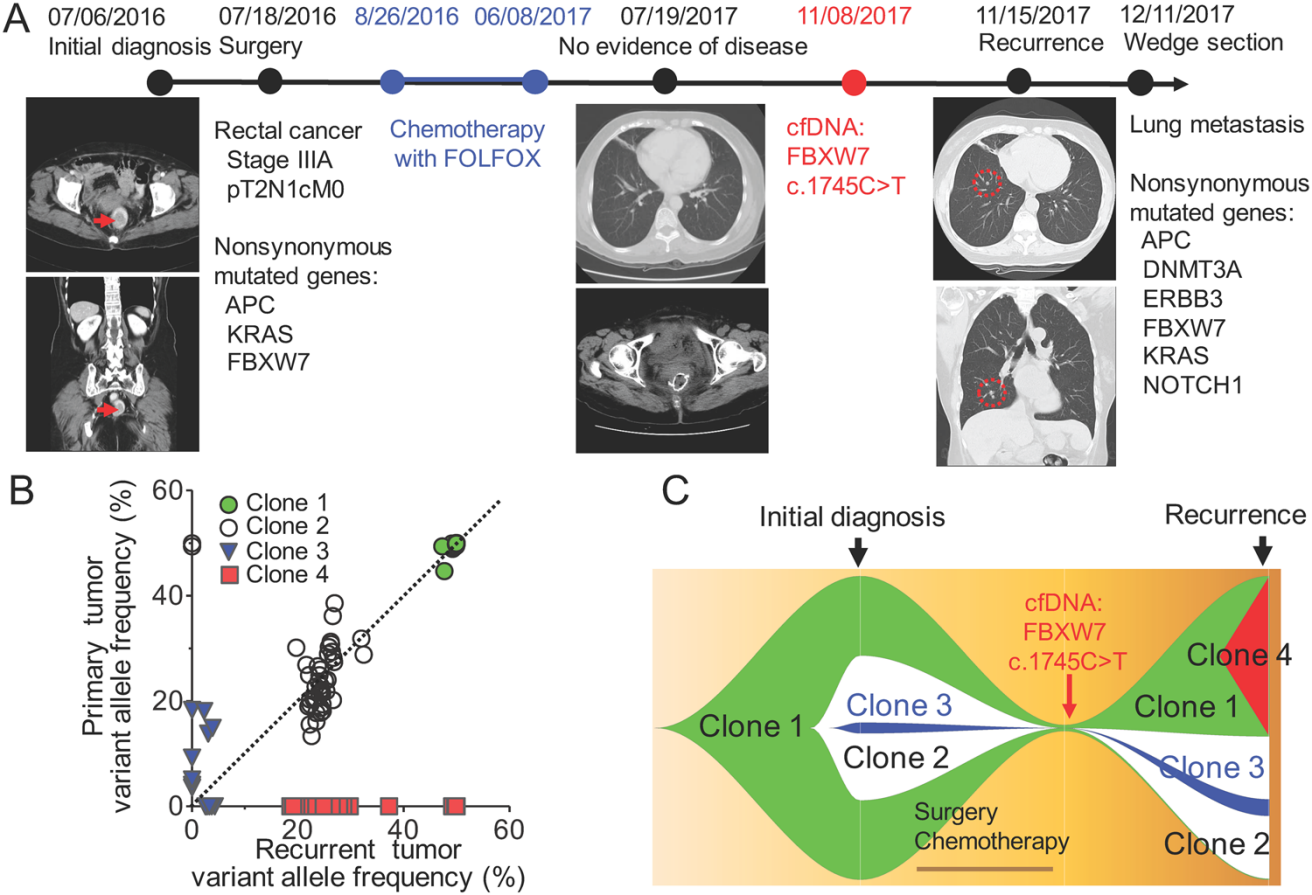
A case study showing the clonal evolution and potential drug targets during cancer progression. (**A**) The clinical time course for a 66-year-old woman with pathological stage III rectal cancer at initial diagnosis. Serial genome sequencing and CT scan were tested. Nonsynonymous recurrent-specific mutated genes including *DNMT3A, ERBB3, NOTCH1* were detected in recurrent tissue. *FBXW7* mutations were detected in primary, recurrent tissue and cell-free DNA. **(B)** The clonal evolution during tumor progression. The variant allele frequency and clustering of variants with similar cellular prevalence were used to reconstruct the clonal evolution. **(C)** Visualizing tumor evolution with the fishplot package. The founding clone 1 (cluster 1 mutations) and the subsequent clone 2 (cluster 2 mutations) and clone 3 (cluster 3 mutations) were all detected in the recurrent tumor. Emerging *NOTCH1* variant was detected in the clone 3 of recurrent tumor tissue. *FBXW7* c.1745C > T was detected in cell-free DNA before the clinical evidence of recurrence.

Figure 4 illustrates a case study showing that Notch signaling pathway might be an important therapeutic target for stage III CRC patients in adjuvant setting. This case is the patient number 5 (Table 2 and Supplementary Table 5), a 66-year-old woman with pathological stage III rectal cancer at initial diagnosis. She did not carry any germline cancer-associated variants and tumor targeted gene sequencing showed somatic mutations in *APC*, *KRAS*, and *FBXW7* in the primary tumor sample. She received standard surgical resection followed by adjuvant chemotherapy with mFOLFOX6 for 12 cycles. No disease recurrence was detected by computed tomography (CT) scan 12 months after surgery. However, when cell-free circulating DNA (cfDNA) was isolated 16 months after surgery, analysis of tumor-specific mutations showed the detection of *FBXW7* c.1745C > T mutation and subsequent CT scan confirmed the recurrence with pulmonary metastasis. Wedge resection of pulmonary metastasis was performed under the curative intent. Three new somatic mutations, including *ERBB3*, *DNMT3A* and *NOTCH1* mutations were detected in the metastatic tumor.

Understanding the clonal evolution during cancer progression offers the insight into tumor heterogeneity and potential drug targets. The variant allele frequency and clustering of variants with similar cellular prevalence were used to reconstruct the clonal evolution^32^. In this case, there are 3 clusters of tumor clones identified in the primary tumor (Fig. 4B,C). The clonal evolution model revealed that clone 1 was the founding clone and the clone 2 and 3 were subsequently derived from clone 1. By contrast, 4 clusters of clones were identified in the recurrent tumor tissues and the clone 3 still existed with additional *NOTCH1* mutation. The detection of *FBXW7* mutation by cfDNA during post-operative follow-up and the emerging *NOTCH1* mutation in the recurrent tumor samples highlight that Notch signaling pathway might be an important therapeutic target for stage III CRC patients in adjuvant setting.

## Discussion

With the advances of NGS technologies, germline WGS is becoming an affordable tool in health care. Here, we demonstrate the application of germline WGS in the care of cancer patients and the impact of germline genetic variants on clinical outcome and therapeutic strategies. Our results highlight the following important points: (i) Germline variants of ACMG SF genes are associated with life threatening toxicities during chemotherapy. (ii) Germline variants of cancer-associated genes affect clinical outcome and screening for the variants should take the ethnicity into consideration. (iii) Notch signaling pathway might be a therapeutic target for CRC patients without germline cancer-associated variants in adjuvant setting. These findings suggest analyzing germline variants with oncologic and non-oncologic phenotypes has a great impact on the care planning of cancer patients.

By comparing the ACMG SF v2.0 gene list, we identified 7 genetic variants with non-oncologic phenotypes in 9 of 159 cancer patients (5.6%). Most of the genetic variants were associated with cardiovascular diseases. For example, *KCNH2*, a genetic variant associated with long QT syndrome 2, was detected in an endometrial cancer patient who developed QT prolongation after chemotherapy with paclitaxel. Genetic susceptibility of chemotherapy-induced cardiac toxicity has been extensively reviewed^33^. Based on the existing evidence, genetic testing before the initiation of treatment to predict risk and reduce the occurrence of cardiotoxicity is recommended. Our result suggests that the *KCNH2* genetic variant is a potential risk factor for paclitaxel-induced QT prolongation. More studies are needed to confirm the predictive role of *KCNH2* in chemotherapy-induced cardiac toxicity.

To identify the germline variants of cancer-associated genes and interpret the clinical significance accurately is important for both cancer patients and their family. By comparing to the pathogenic variants of cancer-associated genes in dbGaP database, we identified 3 germline variants which had relatively high frequency in cancer patients as well as normal subjects, including *PRSS1* c.161 A > G, *SLC22A18* c.257 G > A, and *RAD54B* c.1778A > G. The high frequency in normal Taiwanese population indicates the pathogenicity of these variants for cancer was less likely. This indicates that screening for cancer-associated genetic variant should take the ethnicity into consideration. Interestingly, we found that most of the cancer-associated genes harboring germline mutation were involved in DNA repair, such as *MLH1*, *BRCA1*, *BRCA2*, *MUTYH*, *ATM*, *PMS2*, *MSH6*, *BAP1*, and *FANCA*. Mutations in mismatch repair genes, including *MLH1*, *MSH2*, *MSH6*, and *PMS2*, were the well-known causative germline predisposition factors in CRCs. Recently, the detection of pathogenic variants in Fanconi anemia DNA damage repair pathway genes, such as *BRCA2*, *BRIP1*, *FANCC*, and *FANCE* was also reported in familial CRCs^34^. These results show that defects in DNA repair pathways contribute to carcinogenesis of CRCs. Targeting DNA repair pathways, such as PARP inhibitors, may provide benefits for CRC patients with mutations in DNA repair genes. In this study, 12.5% of colorectal cancer (CRC) patients and 18.2% of gynecological (ovarian or endometrial) cancer patients carried pathogenic germline variants. In general, the prevalence of germline cancer susceptibility gene mutations was about 8% among cancer patients, based on a study analyzing 33 types of cancers from 10,389 cases^35^. However, the prevalence and spectrum of gene mutations vary in the different types of cancers. For example, of 450 patients with early-onset CRC, 16% had germline cancer susceptibility gene mutations^36^. In addition, 19.9% of ovarian cancer patients have been reported carrying the pathogenic germline mutation^35^. The impact of VUSs on genomic function was considered from different perspectives, such as cancer enrichment, LOH, protein expression and other evidence^35^. In this study, there were 3 patients with cancer family history carrying VUSs. These VUSs were not detected in the reference group of Taiwan BioBank. We thus considered these VUSs as potentially pathogenic variants^37^.

After surgical resection, adjuvant chemotherapy with mFOLFOX is the standard treatment for stage III CRC patients. The 5-year disease-free survival rate is around 73%. A lot of clinical studies have tried to further improve the outcome of these patients. However, no progress was achieved. Here we found CRC patients without germline cancer-associated variants had a worse outcome than those with germline variants. Mutations of *FBXW7* and *NOTCH1*, which could lead to aberrant activation of Notch signaling pathway, were only detected in patients without germline cancer-associated variants. In addition, the analysis of clonal evolution also revealed *NOTCH1* mutation was the most common somatic mutation detected in recurrent tumor samples. The important roles of *NOTCH1* signaling in CRC have been well studied and it has been shown that *NOTCH1* mutations could lead to aberrant activation of Notch signaling pathway contributing cancer behaviors^38,39^. Here we identified *NOTCH1* mutation was the most common somatic mutation detected in recurrent CRC samples. We have checked the *NOTCH1* mutations in Integrative Genomics Viewer to rule out technical artefacts and the pathogenicity was assessed by the variant scoring systems, such as SIFT and PolyPhen^40^. The *NOTCH1* mutation detected in this study (p.Gly347Asp and p.His727Tyr) was predicted to affect the protein function. Further study on the biological impact of individual *NOTCH1* mutations will be evaluated in the future. These results suggest Notch signaling pathway might be a potential therapeutic target for stage III CRC patients in adjuvant setting.

Using unbiased analysis to identify the association between uncharacterized variant and cancer susceptibility or prognosis is an interesting topic that we are working on. The major aim of this study is to investigate whether germline genetic variants have an impact on clinical outcome or therapeutic strategy. That’s why we comprehensively analyzed the germline genetic variants by WGS and used the established database to identify variants that are known to be implicated in cancer susceptibility, drug response and non-oncogenic phenotypes.

Despite the use of established database, our study showed several important findings. The first is the underlying germline genetic alterations could mold the tumor mutational landscape. The frequency of several tumor mutational signatures, including signature 1B, 11, 12 and 19, was significantly different between CRC patients with and without germline cancer-associated genetic variants. In addition, the somatic mutations detected in patients with and without germline cancer-associated variants were different, too. These results imply that the therapeutic strategies should be tailored by both germline background and somatic mutations. Second, the pathogenicity of several cancer-associated genetic variants, including *PRSS1* c.161A > G, *SLC22A18* c.257G > A, and *RAD54B* c.1778A > G, was misclassified. The frequency of the above genetic variants in the reference population was high (32.46, 12.42 and 3.01%, respectively). High allele frequency in population data is a strong evidence of benign impact^41^. Our results provide the new evidence of misclassification for these 3 germline genetic variants and suggest that the ethnic difference should be taken into consideration to minimize false-positive results. Third, 6% of cancer patients presented the secondary findings of germline variants with non-oncogenic impact, mainly on the cardiovascular system. We demonstrated abnormal cardiac electricity developed in a patient carrying the variant in *KCNH2* while receiving the adjuvant chemotherapy. The cardiac abnormality became irreversible after the completion of chemotherapy. This result suggests that patients with germline genetic variants with non-oncogenic impact should be carefully monitored during chemotherapy.

In conclusion, this study highlights the importance of applying integrative genomic information for healthcare planning and monitoring side effects during cancer treatment. Germline sequence data and tumor genome have to be analyzed together to have a more complete picture of the overall genetic foundation of cancer.

## Supplementary information

Supplementary information

Supplementary Table 2

Supplementary Table 3

Supplementary Table 4

Supplementary Table 5
