# Author Correction: Germline susceptibility variants impact clinical outcome and therapeutic strategies for stage III colorectal cancer

**DOI:** 10.1038/s41598-020-69145-1

**Published:** 2020-07-17

**Authors:** Peng-Chan Lin, Yu-Min Yeh, Pei-Ying Wu, Keng-Fu Hsu, Jang-Yang Chang, Meng-Ru Shen

**Affiliations:** 1 Department of Internal Medicine, National Cheng Kung University Hospital, College of Medicine, National Cheng Kung University, Tainan, Taiwan; 2 Graduate Institute of Clinical Medicine, National Cheng Kung University, Tainan, Taiwan; 3 Department of Obstetrics and Gynecology, National Cheng Kung University, Tainan, Taiwan; 4 Department of Computer Science and Information Engineering, College of Electrical Engineering and Computer Science, National Cheng Kung University, Tainan, Taiwan; 5 National Institute of Cancer Research, National Health Research Institutes, Tainan, Taiwan; 6 Department of Pharmacology, National Cheng Kung University Hospital, College of Medicine, National Cheng Kung University, Tainan, Taiwan

Correction to: *Scientific Reports* 10.1038/s41598-019-40571-0, published online 08 March 2019

In the original version of this Article, Peng-Chan Lin, Yu-Min Yeh & Jang-Yang Chang were incorrectly listed as being affiliated with ‘Department of Internal Medicine, National Cheng Kung University, Tainan, Taiwan’. The correct affiliation is listed below:

Department of Internal Medicine, National Cheng Kung University Hospital, College of Medicine, National Cheng Kung University, Tainan, Taiwan

Additionally, the Supplementary Information file contained errors in the affiliation list, whereby the affiliations listed were incomplete. These errors have now been corrected in the PDF and HTML versions of the Article and in the accompanying Supplementary Information file.

