# Supplementary material for: Germline susceptibility variants impact clinical outcome and therapeutic strategies for stage III colorectal cancer
Source: Sci Rep. 2019 Mar 8;9:3931. doi: 10.1038/s41598-019-40571-0 (PMC6408483; doi:10.1038/s41598-019-40571-0)
Supplement: Supplementary file 1 — Supplementary information [file 41598_2019_40571_MOESM1_ESM.pdf]

Germline susceptibility variants impact clinical outcome and therapeutic strategies for stage III colorectal cancer

Peng-Chan Lin[1,4], Yu-Min Yeh[1,2], Pei-Ying Wu[3], Keng-Fu Hsu[3],

Jang-Yang Chang[1,5], Meng-Ru Shen[3,6]

[1]Department of Internal Medicine, [2]Graduate Institute of Clinical Medicine, [3]Department of Obstetrics and Gynecology, [6]Department of Pharmacology, National Cheng Kung University Hospital, College of Medicine, National Cheng Kung University, Taiwan; [4]Department of Computer Science and Information Engineering, College of Electrical Engineering and Computer Science, National Cheng Kung University; [5]National Institute of Cancer Research, National Health Research Institutes, Taiwan

Supplementary information

Supplementary Figure S1. Workflow for variant discovery and ClinVar annotations. Detailed description in Materials and Methods.

Supplementary Figure S2. Patients with or without cancer-associated genetic variants and their family cancer history pedigrees.

Supplementary Figure S3. Mutational signature in patients with cancer-associated genetic variants (CAG(+)) or without cancer associated genetic variants (CAG(-)).

Supplementary Table 1. Patients' clinical characteristics.

Supplementary Table 2. ACMG SF v2.0 genetic variants in cancer patients and normal subjects.

Supplementary Table 3. Ethnic-specific genetic variants in cancer patients and normal subjects.

Supplementary Table 4. Germline cancer-associated genetic variants in cancer patients.

Supplementary Table 5. Primary and recurrent tumor mutation variant allele frequency (VAF) and clusters.

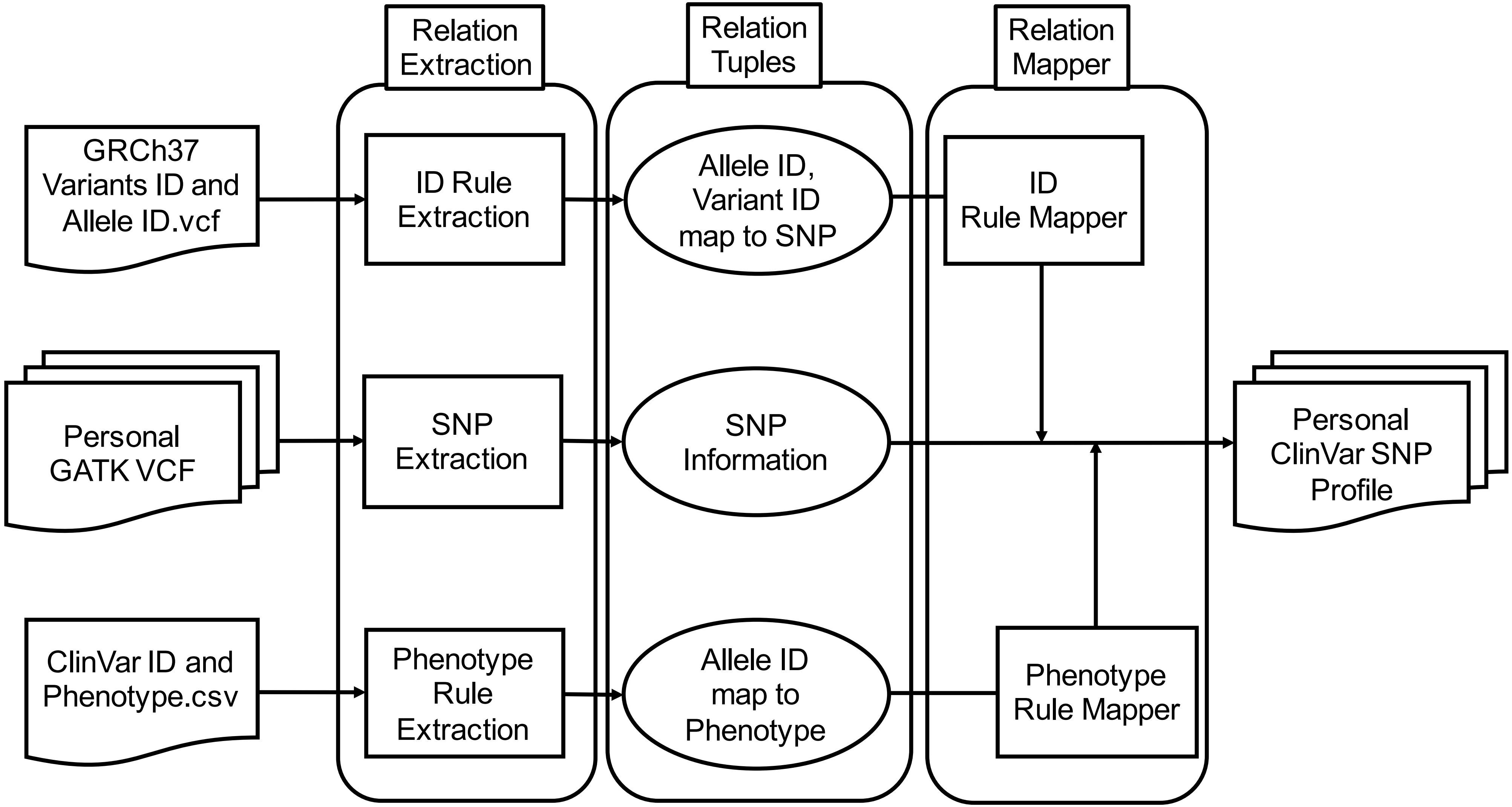

Supplementary Figure S1. Workflow for variant discovery and ClinVar annotations. Detailed description in Materials and Methods.

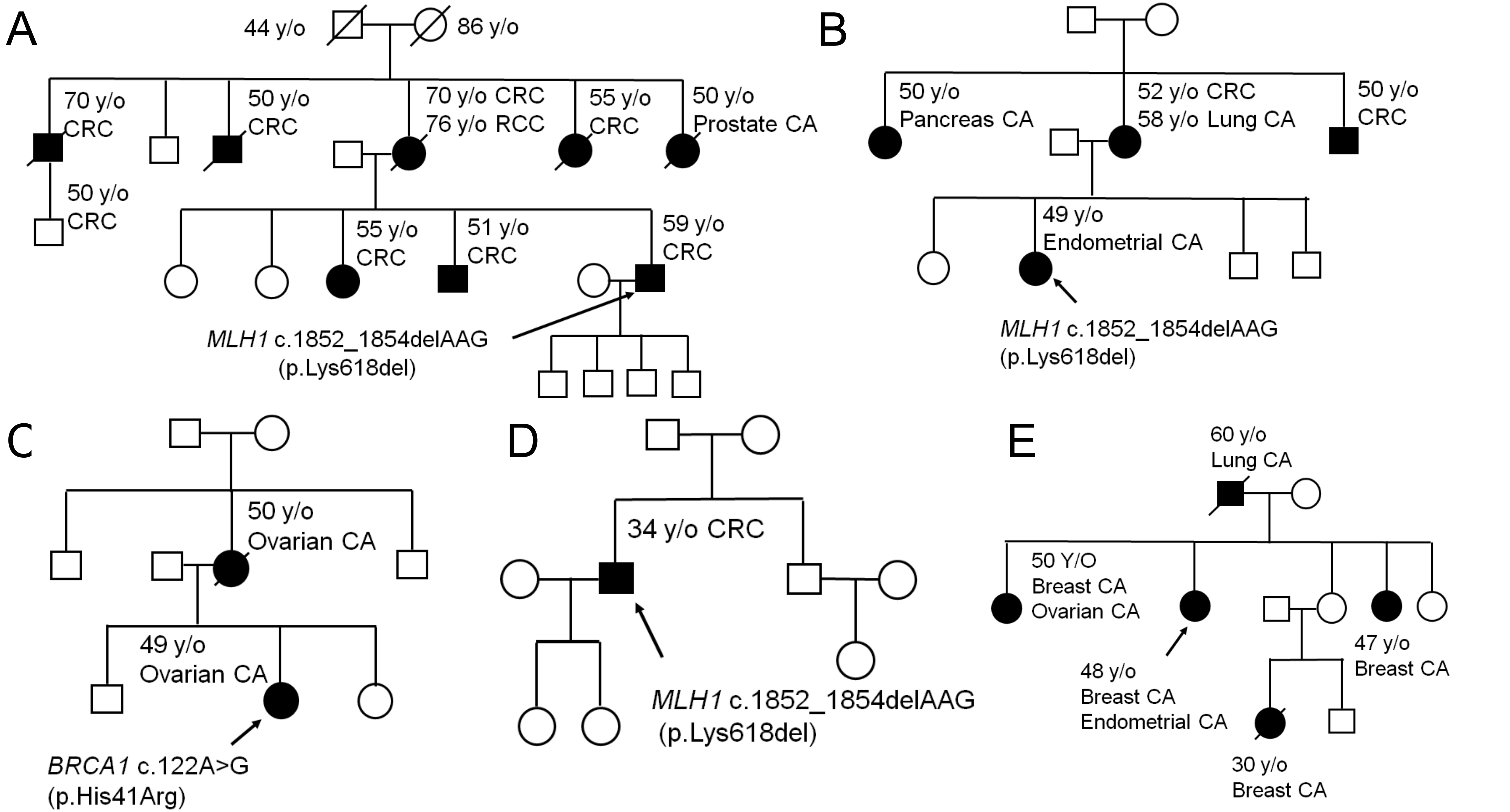

Supplementary Figure S2. Patients with or without cancer-associated genetic variants and their family cancer history pedigrees. Lynch syndrome was diagnosed in (A) one CRC patient and (B) one endometrial cancer patient. Both patients had a germline pathogenic variant of MLH1 c.1852_1854delAAG. (C) A germline pathogenic variant of BRCA1 c.122A>G was detected in one ovarian cancer patient. (D). A MLH1 pathogenic variant was detected in one CRC patient without cancer family history. (E) No well-known cancer-associated genetic variants is identified. Arrows indicate the patients enrolled in this study. A square represents a male and a circle represents a female. A solid square or circle indicates the person has cancer.

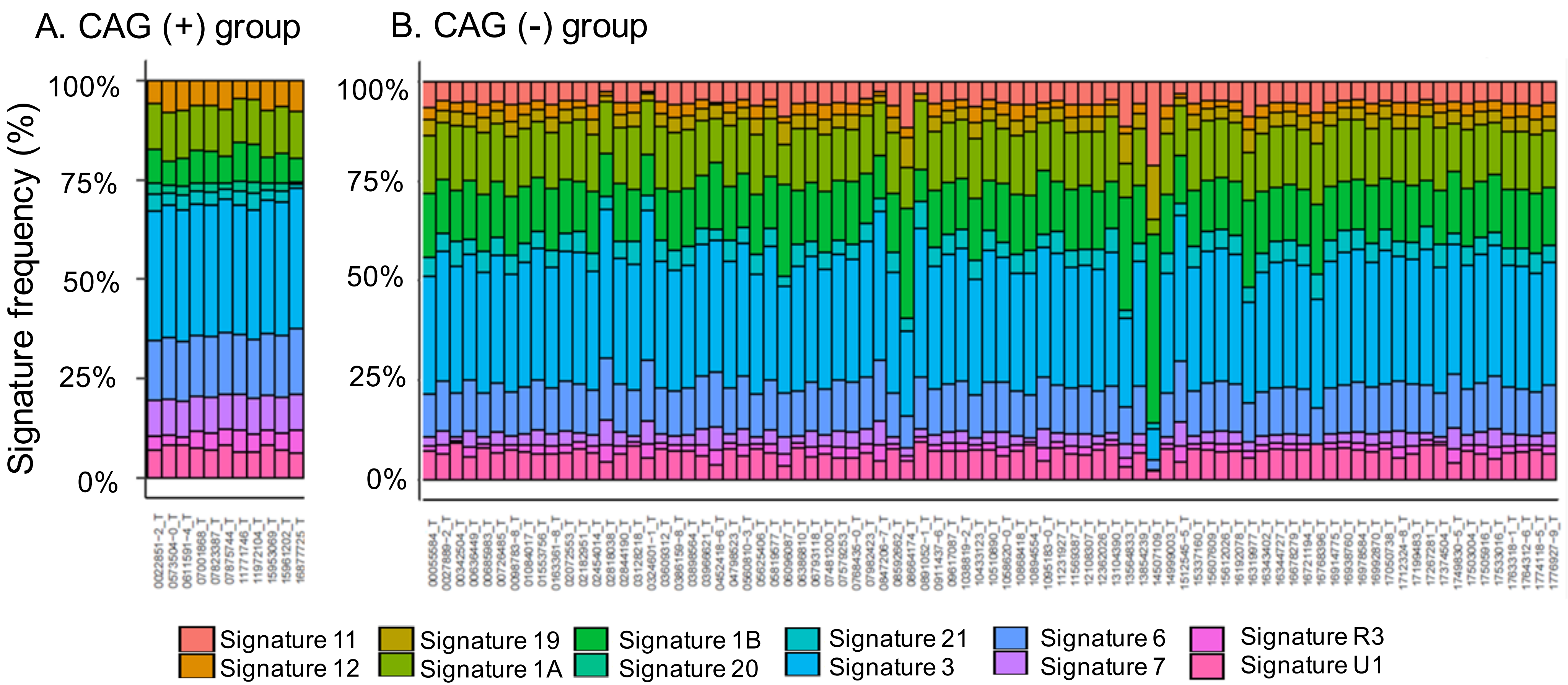

Supplementary Figure S3. Mutational signature in patients with cancer-associated genetic variants (CAG(+)) or without cancer associated genetic variants (CAG(-)). Mutational signature frequency (y-axis) in individual cancer patients (x-axis). Each color indicates a different mutational signature. Signatures 1B, 11, and 19 are predominant in patients without cancer-associated genetic variants.

Supplementary table 1. Patients' clinical characteristics

Characteristics	Patients number (%)
Total	159 (100)
Median age, y/o (range)	57 (23-82)
Gender	
Male	54 (34)
Female	105 (66)
Tumor type	
Colorectal	104 (65.4)
Ovarian	31 (19.5)
Endometrial	24 (15.1)
Cancer family history	
No relatives	77 (48.4)
First-degree relatives	63 (39.6)
Second-degree relatives	19 (11.9)
